# Primary cutaneous CD4+ small/medium T-cell lymphoproliferative disorder. Report of three cases^؆^

**DOI:** 10.1016/j.abd.2026.501307

**Published:** 2026-03-23

**Authors:** Pablo Vargas-Mora, Gonzalo Vera-Santis, David Godoy-Sánchez, Mathias Yagnam-Díaz, Amaranta Luzoro-Vial, Karol Baksai-Elespuru

**Affiliations:** a Dermatology Department, Faculty of Medicine, University of Chile, Santiago, Chile; b Melanoma and Skin Cancer Unit, Instituto Nacional del Cáncer, Santiago, Chile; c Instituto Oncológico Fundación Arturo López Pérez, Organisation of European Cancer Institutes, Santiago, Chile; d Dermatology Department, University of Chile, Santiago, Chile; e Dermatology Service, Clínica MEDS, Santiago, Chile; f Pathology Service, Clínica Las Condes, Santiago, Chile

Dear Editor,

The Primary Cutaneous CD4+ Small/Medium T-cells Lymphoproliferative Disorder (PCSM-LPD) is a clonal proliferation derived from follicular helper T cells, characterized by a predominance of pleomorphic CD4+ T-cells of small to medium size.^1^ It accounts for 2%‒3% of all primary cutaneous lymphomas. The reported age range is 19 to 95 years, with no predilection for race or sex.^2^

In 2016, the WHO-EORTC classification changed the term from lymphoma to lymphoproliferative disorder due to its uncertain potential for malignancy.^1^, ^3^ This condition is rare and frequently underdiagnosed due to low clinical suspicion, which often results in diagnostic errors, unnecessary investigations, and overly aggressive treatments, despite its complete response to conservative therapies. We describe three cases of this rare entity. Also, our series of cases occurred at atypically described ages.

## Case 1

**Fig. 1:**
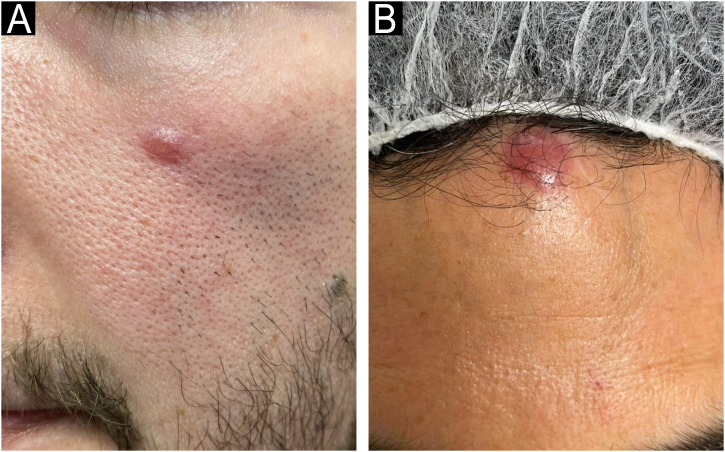
Clinical spectrum of the primary cutaneous CD4+ small/medium T-cell lymphoproliferative disorder. Different clinical presentations of this disorder (A‒B).

A previously healthy 26-year-old male with an asymptomatic pink nodular lesion on the left cheek evolving over six months (Fig. 1A).

## Case 2

A previously healthy 38-year-old female presented with a frontal pink nodule present for three months (Fig. 1B).

## Case 3

**Fig. 2:**
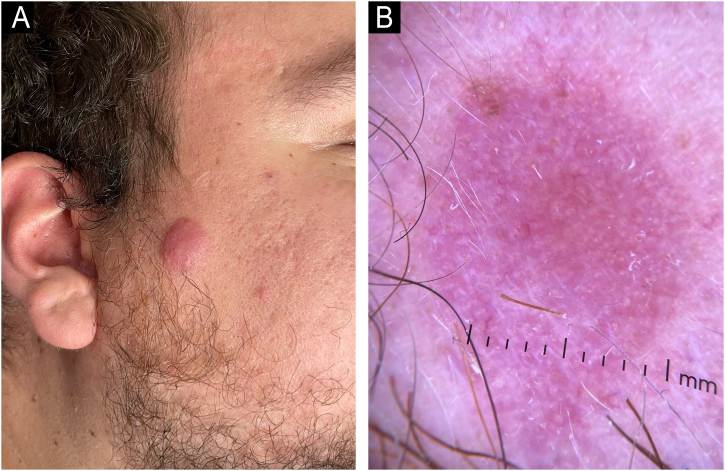
Clinical (A) and dermoscopy (B) findings of case 3 with a diagnosis of primary cutaneous CD4+ small/medium T-cell lymphoproliferative disorder.

A previously healthy 27-year-old male reports a pink nodule in the right preauricular region with a four-month history (Fig. 2A).

**Fig. 3:**
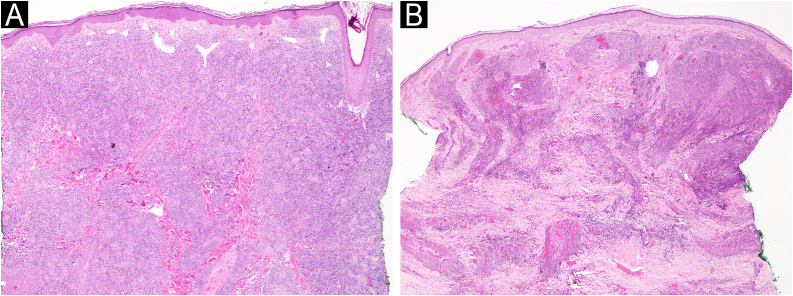
Histopathology of the primary cutaneous CD4+ small/medium T-cell lymphoproliferative disorder. The lymphoid cells are small to medium-sized in the Hematoxylin and Eosin staining. (A‒B).

**Fig. 4:**
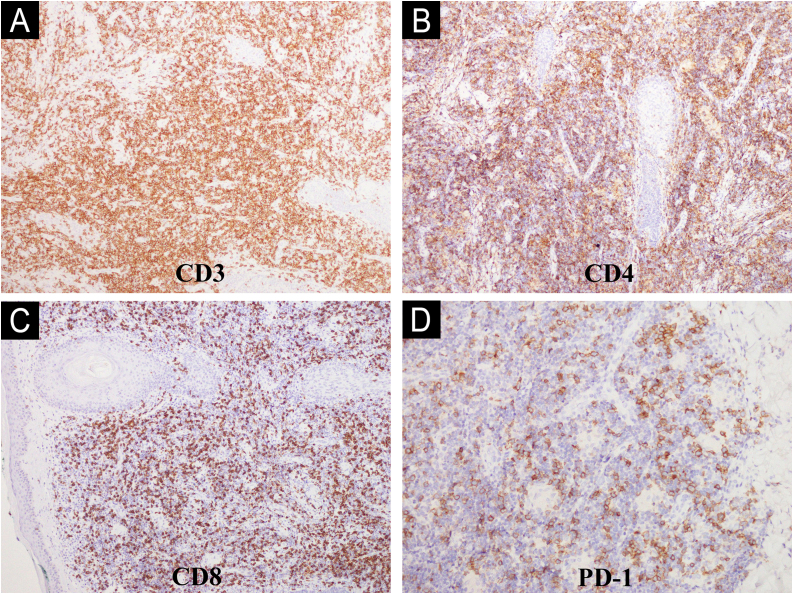
Immunohistochemical markers of the primary cutaneous CD4+ small/medium T-cell lymphoproliferative disorder. The lymphoid cells mostly show the CD3+, CD4+, CD8+ and PD-1 phenotype (A, B, C and D respectively).

In all three cases, the lesions were asymptomatic and clinically similar to one another, with nonspecific dermatoscopy findings characterized by homogeneous pinkish-salmon-colored lesions with white comedo-like dots (Fig. 2B). Regarding histopathology, the cases exhibited a small to medium-sized lymphocyte infiltrate with a nodular pattern and mild to moderate atypia (Fig. 3 A and B). Immunohistochemistry revealed positivity for CD3, CD4, CD8, and PD1 markers (Fig. 4 A‒D), while Bcl-6, CD7, and CD30 were negative. The Ki-67 proliferation index ranged from 10% to 30%. Clonality testing was not performed in any of the cases, as the clinicopathological correlation was consistent with the diagnosis.

In the male cases, there was complete spontaneous resolution after 2- and 3-months following the incisional biopsy, respectively. In the female case, the biopsy was excisional, and no further management was required. No recurrence was observed in any of these cases after one year of follow-up.

The PCSM-LPD can occur at any age, but it most commonly affects individuals in their sixth to seventh decade of life.^4^, ^5^, ^6^ Between 1992 and 2021, Plumptre et al. evaluated 209 cases of PCSM-LPD from 38 published studies and reported a mean age at diagnosis of 55 years.^2^

This disorder typically presents as a single, pink, asymptomatic tumor or plaque located on the face or neck. It can appear acutely and exhibit rapid growth, or it may have a slow progression over years with minimal changes or even resolve spontaneously.^3^ Atypical presentations have been reported, including alopecic plaques, poikilodermatous plaques, pigmented purpuric-like lesions, acneiform lesions, and a clinical association with elastolytic giant cell granuloma.^3^, ^7^

This entity is histopathologically characterized by nodular and/or diffuse infiltrates of small to medium-sized pleomorphic T-lymphocytes, positive for CD3 and CD4, primarily localized in the superficial dermis. These infiltrates may extend into the deep dermis or even the subcutis. They may exhibit epidermotropism, although it is focal rather than band-like, as seen in mycosis fungoides. The proliferative index Ki-67 is typically low (5%–30%).^4^, ^5^ Immunohistochemical markers are generally negative for CD8 and CD30, though studies have identified CD30+ lymphocytes in 15%–30% of cases. Loss of CD7 is classically described in up to 84% of cases.^4^

The presence of certain follicular markers such as PD-1 (CD279), Bcl-6, and CXCL13 was initially described as a diagnostic criterion; however, subsequent studies have demonstrated that these markers can also be found in other primary cutaneous lymphomas.^3^, ^6^

In some cases, a subset of large, atypical, pleomorphic cells may be present, but by definition, they should constitute less than 30% of the infiltrate. A higher proportion would be more consistent with a diagnosis of primary cutaneous Peripheral T-cell Lymphoma, Not Otherwise Specified (PTCL-NOS), rather than PCSM-LPD. This is clinically relevant, given the markedly worse prognosis, with a 5-year survival of less than 20%.^4^, ^5^

Monoclonal rearrangements of the T-Cell Receptor (TCR) genes have been identified in approximately 60% to 100% of reported cases,^6^, ^8^ Beltraminelli et al. conducted the largest series with 124 patients and reported only a 60% prevalence of TCR-gamma gene rearrangements,^8^ whereas Alberti-Violetti et al. reported an 84% prevalence, representing the second largest series.^4^ Despite these findings, TCR gene clonality studies are not essential for diagnosis. Their utility is primarily reserved for cases in which the histopathological features are inconclusive or when a cutaneous lymphoid hyperplasia is suspected.^3^ When clinical and histopathological findings are concordant, clonality testing is generally unnecessary. Specialty neither these immunohistochemical criteria nor demonstration of clonality is specific for malignant lymphomas.^9^

The differential diagnoses include nodal peripheral T-cell lymphoma, non-otherwise specified, pseudolymphomas, tumor-stage mycosis fungoides, and primary cutaneous B-cell lymphomas.^3^, ^4^, ^5^

Regarding treatment of solitary lesions, expectant management may be appropriate given the potential for spontaneous resolution. Other treatments include surgery, intralesional corticosteroids, local radiotherapy, and phototherapy, among others.^10^ In cases of refractory solitary lesions or multiple lesions, therapies such as PUVA or narrowband UVB phototherapy, oral doxycycline, interferon-α, cyclophosphamide, or combination chemotherapy regimens such as CHOP have been described.^10^

The PCSM-TLD has a very favorable prognosis, with a 5-year survival rate of 80%–90% in cases with solitary lesions^1^, ^7^ and 60%‒80% in those with multiple lesions. However, the lower survival in the latter group may be explained by the inclusion of patients diagnosed with NOS lymphomas or primary cutaneous follicular helper T-cell lymphomas.^3^, ^4^

The PCSM-LPD is a rare and indolent condition. Despite its histopathological appearance, it has an excellent prognosis and may resolve spontaneously. Recognizing its clinical and histopathological features is essential to avoid misdiagnosis and aggressive treatments. However, this requires an accurate diagnosis based on thorough histopathological and immunophenotypic analysis, as misdiagnosis may occasionally lead to delayed treatment of an aggressive lymphoma.

## ORCID ID

Pablo Vargas-Mora: 0000-0002-9388-2940

David Godoy-Sánchez: 0000-0003-4809-5216

Mathias Yagnam-Díaz: 0009-0007-6672-8482

Amaranta Luzoro-Vial: 0009-0003-1050-1925

Karol Baksai-Elespuru: 0009-0007-6065-1218

## Financial support

None declared.

## Authors' contributions

Pablo Vargas Mora: Contributed to the collection of clinical and research data. Intellectual participation in the therapeutic conduct of some of the reported cases. Also participated in the review of the literature and approved the final manuscript prior to submission.

Gonzalo Vera Santis: Contributed to the collection of clinical and research data. Also participated in the review of the literature and approved the final manuscript prior to submission.

David Godoy Sánchez: Contributed to the collection of clinical and research data. Intellectual participation in the therapeutic conduct of some of the reported cases. Also participated in the review of the literature and approved the final manuscript prior to submission.

Mathias Yagnam Díaz: Contributed to the collection of clinical and research data. Intellectual participation in the therapeutic conduct of some of the reported cases. Also participated in the review of the literature and approved the final manuscript prior to submission.

Amaranta Luzoro Vial: Contributed to the collection of clinical and research data. Intellectual participation in the therapeutic conduct of some of the reported cases. Also participated in the review of the literature and approved the final manuscript prior to submission.

Karol Basai Elespuru: Contributed to the collection of clinical and research data. Also participated in the literature review and the intellectual analysis of the histopathological findings of the three cases and approved the final version of the manuscript prior to submission.

## Research data availability

The entire dataset supporting the results of this study was published in this article.

## Conflicts of interest

None declared.
